# *Paraboea dolomitica* (Gesneriaceae), a new species from Guizhou, China

**DOI:** 10.3897/phytokeys.153.50933

**Published:** 2020-07-16

**Authors:** Zhiyou Guo, Zhaowen Wu, Weibin Xu, Zhenyu Li, Xiaoguo Xiang

**Affiliations:** 1 Qiannan Normal College for Nationalities, College of Biological Sciences and Agriculture, Duyun, 558000, China College of Biological Sciences and Agriculture Duyun China; 2 Jiangxi Province Key Laboratory of Watershed Ecosystem Change and Biodiversity, Institute of Life Science and School of Life Sciences, Nanchang University, Nanchang 330031, China Nanchang University Nanchang China; 3 Guangxi Key Laboratory of Plant Conservation and Restoration Ecology in Karst Terrain, Guangxi Institute of Botany, Guangxi Zhuang Autonomous Region and the Chinese Academy of Sciences, Guilin 541006, China Guangxi Institute of Botany Guilin China; 4 State Key Laboratory of Systematic and Evolutionary Botany, Institute of Botany, Chinese Academy of Sciences, Beijing 100093, China Institute of Botany, Chinese Academy of Sciences Beijing China

**Keywords:** Gesneriaceae, limestone flora, new species, *
Paraboea
*

## Abstract

Here we describe *Paraboea dolomitica* Z.Y. Li, X.G. Xiang & Z.Y. Guo, a new species of Gesneriaceae from Guizhou, China. Based on recent extensive observations, this new species is morphologically similar to *Paraboea filipes* (Hance) Burtt, in having obovate leaf blades, 1–4-flowered cymes and purplish corolla, but differs from that species by the combination of denticulate leathery leaves, sparsely brown haired peduncles, two woolly bracts, reniform anthers and two glabrous staminodes. Additionally, molecular data support this new species as a member of a clade that includes *P. crassifolia*, *P. tetrabracteata*, *P. peltifolia*, *P. vetutina*, *P. dushanensis*, *P. dictyoneura*, *P xiangguiensis* and *P. guilinensis*, but it is distinct from them in leaf position, inflorescence, penduncle, bract and capsule. The conservation status of this species is considered to be “Vulnerable” (VU) according to the IUCN Red List Categories and Criteria.

## Introduction

*Paraboea* was published by Clarke (1883) as a section of the *Didymocarpus* Wall. and subsequently treated as a distinct genus by Ridley (1905). Burtt (1984) recircumscribed *Paraboea* based on the indumentum instead of fruit morphology, and many species were transferred to *Paraboea* from the genus *Boea* Comm. ex Lam. Xu et al. (2008) revised this genus and recognised 89 species and five varieties. Using ITS and *trnL*-*F*, a recent molecular phylogenetic study indicated that *Trisepalum* C.B. Clarke and *Phylloboea* Benth. were nested in *Paraboea*, and consequently 15 new combinations in *Paraboea* were made (Puglisi et al. 2011). Further, Puglisi et al. (2016) established a new genus *Middletonia* segregated from *Paraboea*.

To date, *Paraboea* (C.B.Clarke) Ridley contains approximately 142 species and is distributed in southern China, northeastern India, the eastern Himalayas, Burma, Thailand, Cambodia, Laos, Vietnam, Malaysia, Philippines and Indonesia east to Sulawesi, occurring mostly in limestone regions (Xu and Burtt 1991; Xu 1994; Li and Wang 2004; Xu et al. 2008; Chen et al. 2008; Puglisi et al. 2011; Xu et al. 2012; Wen et al. 2013; Xu et al. 2017a; Puglisi and Phutthai 2018). Xu et al. (2017b) summarised that there are ca. 28 species in China, mainly in limestone areas of south and southwest China. Since then, one new species and one new record have been discovered in China (He et al. 2018; Lu et al. 2019). During our expeditions to Wuyang River, Zhenyuan County and Yuntai Mountain, Shibing County, Guizhou, China in 2016 and 2017, an unidentified species of *Paraboea* was collected. Based on morphological and molecular data, we concluded that it is a significant new species, which we describe here.

## Materials and methods

### Morphological observations

Morphological observations and measurements of the new species were carried out, based on living plants in the field and dry specimens in herbarium (PE and QNUN, herbarium acronyms according to Index Herbariorum; Thiers 2020). The photographs were taken in the field. All morphological characters were studied under dissecting microscopes and are described using the terminology presented by Wang et al. (1998).

### Taxon sampling and DNA sequencing

A total of 60 species of *Paraboea* were sampled. Based on Roalson and Roberts (2016) and Xu et al. (2017a), seven species (*Middletonia evrardii* (Pellegr.) C.Puglisi, *Middletonia monticola* (Triboun & D.J.Middleton) C.Puglisi, *Middletonia multiflora* (R.Br.) C.Puglisi, *Isometrum farreri* Craib, *Kaisupeea herbacea* (C.B.Clarke) B.L.Burtt, *Ornithoboea arachnoidea* (Diels) Craib and *Ornithoboea wildeana* Craib) were selected as outgroups. No material of *P. filipes* (Hance) Burtt, the most morphologically-similar species, was available for analysis.

Total genomic DNA was extracted from leaves dried in silica gel using the Plant Genomic DNA Kit (CW Biotech, Beijing, China). The nuclear internal trancribed spacer (ITS) and chloroplast *trnL^UAA^-F^GAA^* (including intron and spacer) were used in this study. The primers for ITS were ITS-5P (5’-GGA AGG AGA AGT CGT AAC AAG G-3’) and ITS-8P (5’-CAC GCT TCT CCA GAC TAC-3’) (Möller and Cronk 1997) and primers for *trnL-F* were *c* (5’-CGA AAT CGG TAG ACG CTA CG-3’) and *f* (5’-ATT TGA ACT GGT GAC ACG AG-3’) (Taberlet et al. 1991). The selected DNA regions were amplified with standard polymerase chain reaction (PCR) and products were analysed by MajorBio company (Beijing, China). Voucher information and GenBank accession numbers are listed in Appendix 1. Except for sequences of the new species that were generated in this study, others are from GenBank.

### Alignment and Phylogenetic analysis

Sequences were aligned using the default parameters in CLUSTAL X v1.83 (Thompson et al. 1997) and manually adjusted with BIOEDIT v5.0.9 (Hall 1999). Phylogenetic analyses were carried out using Maximum Parsimony (MP) and Bayesian Inference (BI) methods in PAUP v4.0b10 (Swofford 2003) and MrBayes v3.2.0 (Ronquist and Huelsenbeck 2003), respectively. For MP analyses, heuristic searches were performed with 1000 random sequence addition replicates, tree-bisection-reconnection (TBR) branch swapping, MulTrees in effect and steepest descent off. Gaps were treated as missing data, characters were equally weighted and their states were unordered. Internal branch support was estimated by using 1000 bootstrap replicates (Felsenstein 1985), as described above. For BI analyses, the nucleotide substitution model was determined by the Akaike Information Criterion (AIC) in Modeltest v3.06 (Posada and Crandall 1998). Four chains of the Markov Chain Monte Carlo (MCMC) were run over 3 million generations, sampling one tree every 1000 generations, starting with a random tree. Majority rule (> 50%) consensus tree was constructed after removing the burn-in period samples (the first 25% of the sampled trees).

## Results

**Figure 1.**
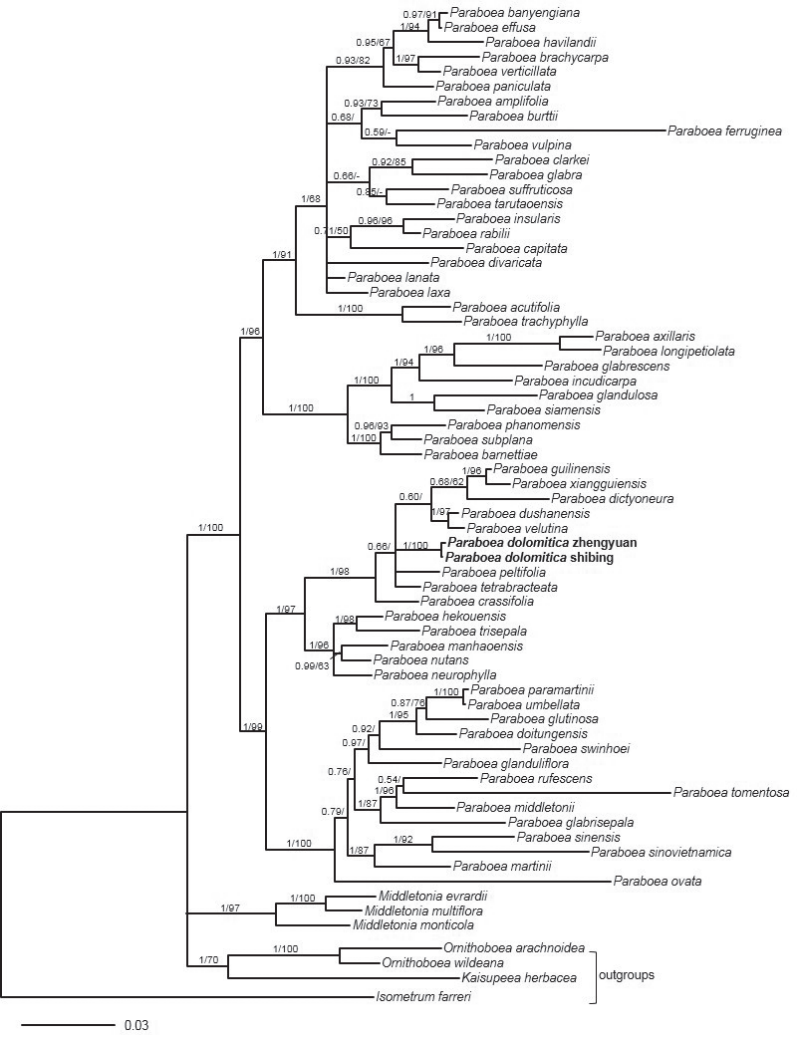
The majority consensus tree of the Bayesian Inference method based on ITS and *trnL-F* regions. Bayesian posterior probabilities and bootstrap support values (> 50%) are shown above the branch. The new species is highlighted in bold.

The concatenated DNA matrix had a length of 1944 aligned characters (ITS: 993 bp and *trnL-F*: 951 bp), of which 838 were variable and 475 are parsimony-informative. MP and BI analyses resulted in congruent topologies except for some clades with low supported values (Fig. 1). The genus *Paraboea* was supported as a monophyletic with strong support values. The major phylogenetic relationships amongst *Paraboea* were consistent with Xu et al. (2017a). The two samples of the new species from different sites are shown as a distinct clade (Posterior Probability (PP) = 1.00, Bootstrap value (BS) = 100%). The new species forms a monophyletic clade with *P. crassifolia*, *P. tetrabracteata*, *P. peltifolia*, *P. vetutina*, *P. dushanensis*, *P. dictyoneura*, *P xiangguiensis* and *P. guilinensis* (PP = 1.00, BS = 98%), but its sister group is uncertain (Fig. 1).

### Taxonomic treatment

#### Paraboea dolomitica

Taxon classificationPlantaeLamialesGesneriaceae

Z.Y. Li, X.G. Xiang & Z.Y. Guo sp. nov.

5EE482F6-1485-5B12-AA8C-89BF6F34461D

urn:lsid:ipni.org:names:77210596-1

Figs 2 , 3

##### Diagnosis.

*Paraboea dolomitica* is morphologically similar to *P. filipes*. Both of them have obovate leaf blades, 1–4-flowered cymes and a purplish corolla, but *P. dolomitica* differs from *P. filipes* by its leathery leaves with denticulate margins (*vs.* papery leaves with subentire margins in *P. filipes*), peduncles sparsely covered with brown hairs (vs. sparsely sericeous-lanate when young and glabrate when mature), two woolly bracts (vs. two glabrous bracts), reniform anthers (vs. oblong anthers), two staminodes 0.3 cm long (vs. 1 staminodes 0.02 cm long), and flowering during April and May (vs. flowering during September and October) (Table 1).

**Figure 2.**
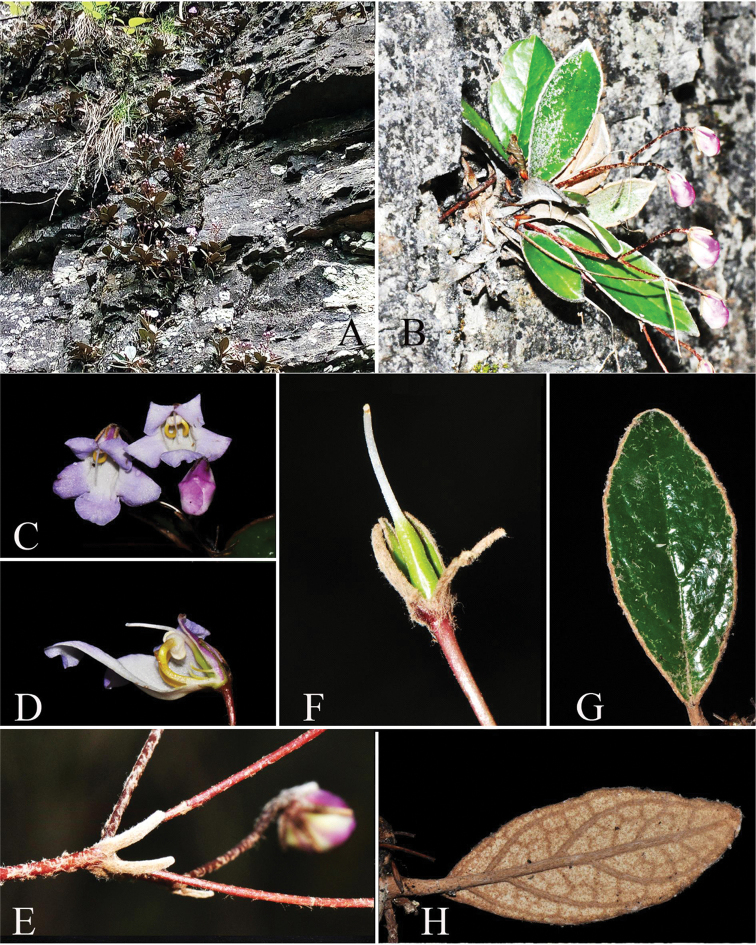
*Paraboea dolomitica*. **A** Habitat **B** flowering habit **C** flower face view **D** opened corolla showing stamens, staminodes and pistil **E** bracts **F** pistil with calyx **G** adaxial leaf blade; and **H** abaxial leaf blade.

Phylogenetic analysis suggested that *P. dolomitica* was nested in a clade including *P. crassifolia* (Hemsl.) Burtt, *P. tetrabracteata* F. Wen, Xin Hong & Y. G. Wei, *P. peltifolia* D. Fang et Z. Zeng, *P. vetutina* (W. T. Wang et C. Z. Gao) Burtt, *P. dushanensis* W. B. Xu & M. Q. Han, *P. dictyoneura* (Hance) Burtt, *P xiangguiensis* W. B. Xu & B. Pan and *P. guilinensis* L. Xu et Y. G. Wei, but *P. dolomitica* can be easily differentiated from them in leaf position, inflorescence, penduncle, bract and capsule. The detailed morphological comparison of the species most morphologically similar to *P. dolomitica* is listed in Table 1.

**Table 1.** Morphological comparisons between *Paraboea dolomitica* and its relatives its relatives based on morphological observation and phylogenetic analyses.

Characters	*P. dolomitica*	*P. filipes*	*P. dictyoneura*	*P. crassifolia*	*P. dushanensis*	*P. peltifolia*
Rhizome	1.5–6.0 cm long, ca. 0.3–0.5 cm diam.	up to 2.5 cm long, ca. 0.3 cm diam.	1.5–2.5 cm long, 0.7–0.8 cm diam.	0.5–1.5 cm long, 0.5–0.9 cm diam.	4–10 cm long, 0.2–0.6 cm diam.	2–7 cm long, 0.5–1 cm diam.
Stem	present	absent	absent or up to 10 cm	absent or up to 15 cm	absent	present
Leaf position	crowded near the stem apex, opposite	basal, rosette	basal or crowded near the stem apex, rosette	basal or crowded near the stem apex	congested at the apex of rhizome	spiral at the stem apex
Leaf blade	leathery, obovate to elliptic, 2.5–4.5 × 1.0–1.5 cm, margin denticulate	papery, obovate to obovate-oblong, (1~) 2–5 × (0.3~) 0.7–2.2 cm, margin shallowly crenate or subentire	thick papery, oblanceolate, 7–19 × 1.2–4.5 cm, margin serrate to dentate or subentire	thick papery, obovate or ovate, 3–16 × 1.5–7 cm, margin crenate to denate or subentire	leathery, cuneate to attenuate, 4–8 × 0.7–1.5 cm, margin crenate to shallowly repand	papery, obovate to oblanceolate, spatulate or subspatulate, 6–33.5 × 3–14.3 cm, margin crenate-serrate
Cymes	1–4-flowered	1–4-flowered	5–20-flowered	4–12-flowered	1–5-flowered	2–15-flowered
Peduncle	3–5 cm long, sparsely lanate with glandular hairs	3–7 cm long, glabrescent	8–21 cm long, pannose to sparsely pannose	3–12 cm long, woolly to pannose	3–5 cm long, ferruginous matted indumentum	4–6 cm long, woolly
Bract	2, linear, 0.3–0.4 cm long	2, narrowly oblong-ovate, ca. 0.1 cm long	2 or 3, lanceolate to narrowly oblong, 0.5–1.3 cm long	2, linear to subulate, 0.2–0.5 cm long	2, linear-lanceolate, 0.3–0.5 cm long	2, lanceolate-triangular, 0.2–0.3 (~0.4) cm long
Calyx	5-parted	5-parted	5-parted	5-parted	5-parted	2-lipped
Corolla	purplish	purplish	purplish	purplish	purple-blue	white
Anther	reniform	narrowly oblong	oblong	oblong	elliptic	reniform
Staminodes	2, 0.3 cm long	1, ca. 0.02 cm long	3, 0.2–0.45 cm long	2, 0.2–0.25 cm long	3, 0.25–0.3 cm long	2, 0.2 cm long
Capsule	1.5–1.8 cm long, slightly twisted	0.5–1.1 cm long, not twisted	1.5–6 cm long, spirally twisted to nearly straight	2–4.5 cm long, spirally twisted	1.2–3.1 cm long, not twisted	1–3.6 cm long, not twisted
Flowering	April and May	September and October	April and May	June and July	May and June	March and April

Note: The morphological characters of *P. filipes*, *P. dictyoneura*, *P. crassifolia* and *P. peltifolia* are from Li and Wang (2004) and the characters of *P. dushanensis* are from Xu et al. (2017a).

##### Type.

China. Guizhou: Shibing County, Yuntai Mountain, 27°06'80.7"N, 108°07'00.0"E, elevation 885 m, on rock faces of a karst dolomite cave, 2 May 2017, Z.Y. Guo 20170047 (holotype: PE!; isotypes: PE!, QNUN!).

Perennial herbs. Rhizomes subterete, 1.5–6.0 cm long, 0.3–0.5 cm diam. Roots slender, fibrous. Leaves crowded near stem apex, opposite; blade leathery, obovate to elliptic, 2.5–4.5 cm long, 1.0–1.5 cm wide, apex acute or rounded, base rounded to broadly cuneate, margin denticulate, involute; adaxial surface with arachnoid covering when young, but glabrescent when mature, abaxially densely brown woolly; principal vein depressed above, raised beneath, lateral veins 3–6 on each side of midrib, tertiary venation inconspicuous; petiole 0.8–2.0 cm long, 0.2–0.3 cm broad, densely covered with appressed velvety hairs. Cymes axillary, umbel-like 1–4-flowered; peduncle 3–5 cm long, 0.05–0.08 cm in diameter, sparsely lanate and glandulose-pubescent. Bracts 2, 0.3–0.4 cm long, linear, woolly beneath; pedicel 0.8–2.2 cm long, 0.05–0.1 cm in diameter, sparsely lanate with glandular hairs. Calyx 5-parted, 0.4–0.6 cm long, 0.1–0.15 cm in diameter, apex acute, densely brown woolly; segments linear. Corolla oblique-campanulate, zygomorphic, purplish, 1.0–1.2 cm long, outside and inside glabrous; tube 0.5–0.6 cm long; throat ca. 0.7 cm in diameter; adaxial lip 2-lobed, lobes orbicular or deltoid, abaxial lip 3-lobed, lobes oblong-elliptic or oblong. Stamens 2, glabrous; filaments 0.5–0.6 cm long, ca. 0.08 cm in diameter, yellow, curved at the upper part; anthers reniform, ca. 0.3 cm long, 0.2 cm broad; staminodes 2, linear, ca. 0.3 cm long. Pistil glabrous, ovary linear, stigma capitate. Capsule linear, 1.5–1.8 cm long, 0.15–0.2 cm broad, glabrous, slightly twisted.

##### Distribution.

*Paraboea dolomitica* is known from Yuntai Mountain, Shibing County and Wuyang River, Zhenyuan County, Guizhou, China.

##### Phenology.

Flowering occurs in April and May and the fruiting occurs between June and August.

##### Etymology.

The specific epithet refers to the habitat of this new species, the dolomite karst area.

##### Habitat and ecology.

*Paraboea dolomitica* grows on rock faces of dolomite karst area, at an elevation of ca. 650–855 m. Accompanying plants in the habitat are sparse and include trees, such as *Platycarya strobilacea* Sieb. et Zucc., *Cotinus coggygria* Scop., and herbs such as *Selaginella moellendorfii* Hieron., *Paphiopedilum micranthum* T. Tang et F. T. Wang, *Viola diffusa* Ging., Galium aparine Linn. var. echinospermum (Wallr.) Cuf. and *Carex* sp.

##### Additional collections.

China. Guizhou: Zhenyuan County, Wuyang River, 27°06'80.7"N, 108°07'00.0"E, elevation 650 m, on rock faces, 3 August 2016, Guo ZY, GZY1608721 (PE and QNUN), GZY1608723 (PE and QNUN), GZY1608724 (PE and QNUN).

**Figure 3.**
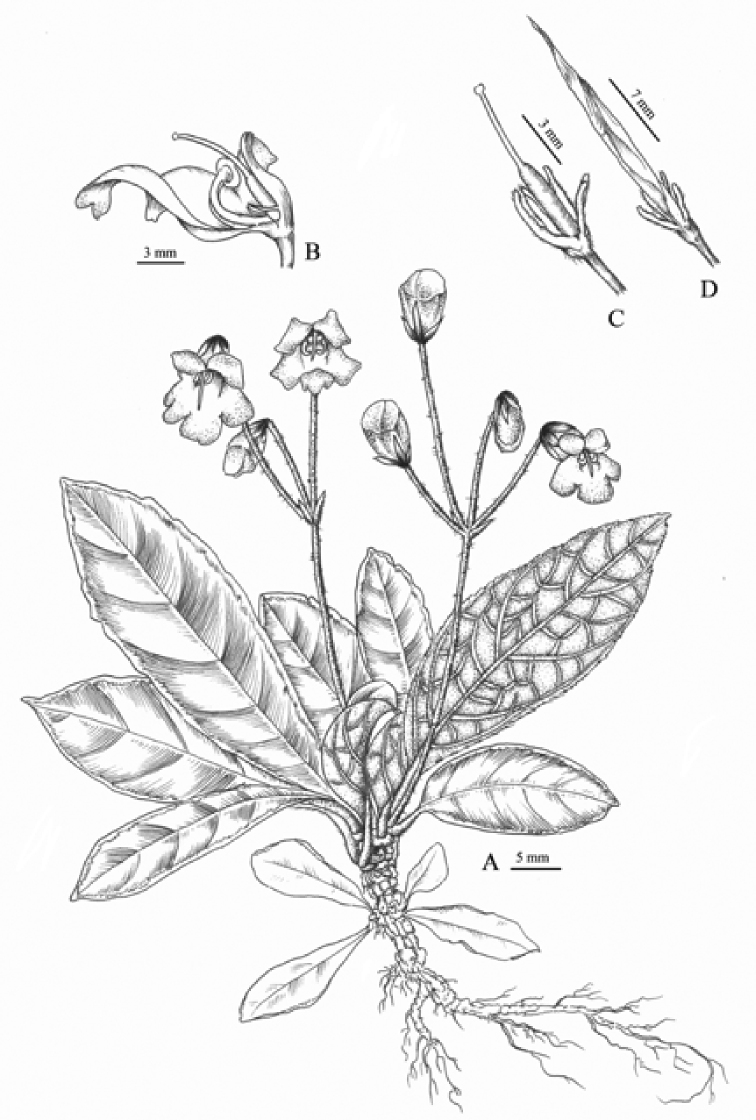
*Paraboea dolomitica*. **A** Flowering habit **B** opened corolla showing stamens, staminode and pistil **C** pistil with calyx and **D** capsule. Drawn by Zhaowen Wu based on holotype and isotypes.

### Proposed IUCN conservation status

The new species has only been found in Shibing County and Zhenyuan County, Guizhou, China. The populations and habitats are vulnerable to human activities such as road construction and deforestation for crops. According to field observations, it has several known populations of less than 300 mature individuals according to field observations. The species is considered to be “Vulnerable” (VUD1) according to the IUCN Red List Criteria (IUCN 2017), based on Criterion D1 and population size, estimated to be fewer than 1000 mature individuals.

## Supplementary Material

XML Treatment for Paraboea dolomitica

## Appendix 1

**GenBank accession numbers (species: voucher, trn *L* - *F* , ITS). The dash indicated that there is no data**

**Ingroups**: *Paraboea acutifolia*, JN934711, FJ501314; *Paraboea amplifolia*, JN934712, JN934754; *Paraboea axillaris*, KU203943, KU203848; *Paraboea banyengiana*, JN934713, JN934755; *Paraboea barnettiae*, AJ492306, KU203847; *Paraboea birmanica*, HQ632866, HQ632958; *Paraboea brachycarpa*, FJ501465, KU203870; *Paraboea burttii*, JN934714, JN934756; *Paraboea capitata*, AJ492298, FJ501315; *Paraboea clarkei*, JN934715, JN934757; *Paraboea crassifolia*, FJ501472, FJ501318; *Paraboea dictyoneura*, FJ501463, KJ475415; *Paraboea divaricata*, JN934717, JN934759; *Paraboea doitungensis*, KU203941, KU203846; ***Paraboea dolomitica***, **Z.Y. Guo 20170047**, MT379849, MT379851; ***Paraboea dolomitica*, GZY 1608721**, MT379850, MT379852; *Paraboea dushanensis*, MF358716, MF358698; *Paraboea effusa*, JN934718, JN934760; *Paraboea ferruginea*, FJ501471, KU203862; *Paraboea glabra*, JN934719, JN934761; *Paraboea glabrescens*, JN934743, JN934785; *Paraboea glabrisepala*, JN934720, JN934762; *Paraboea glanduliflora*, JN934721, JN934763; *Paraboea glandulosa*, HQ632867, JN934784; *Paraboea glutinosa*, JN934722, JN934764; *Paraboea guilinensis*, MF358717, MF358701; *Paraboea havilandii*, JN934724, JN934766; *Paraboea hekouensis*, KU203938, KU203843; *Paraboea incudicarpa*, JN934725, JN934767; *Paraboea insularis*, KU203952, KU203857; *Paraboea lanata*, FJ501467, –; *Paraboea laxa*, FJ501466, –; *Paraboea longipetiolata*, KU203946, KU203851; *Paraboea martinii*, MF358718, MF358702; *Parabora manhaoensis*, KU203937, KU203842; *Paraboea middletonii*, KU203940, KU203845; *Paraboea neurophylla*, JN934727, JN934769; *Paraboea nutans*, MF358719, MF358703; *Paraboea paniculata*, JN934728, JN934770; *Paraboea paramartinii*, JN934729, JN934771; *Paraboea peltifolia*, MF358720, –; *Paraboea phanomensis*, KU203950, KU203855; *Paraboea rabilii*, KU203951, KU203856; *Paraboea rufescens*, JN934730, JN934772; *Paraboea siamensis*, KU203948, KU203853; *Paraboea sinensis*, JN934731, JN934773; *Paraboea sinovietnamica*, MF358722, MF358706; *Paraboea subplana*, JN934744, JN934786; *Paraboea suffruticosa*, JN934732, JN934774; *Paraboea swinhoei*, FJ501475, JN934775; *Paraboea tarutaoensis*, JN934734, JN934776; *Paraboea tetrabracteata*, MF358723, MF358707; *Paraboea tomentosa*, KU204043, KU203971; *Paraboea trachyphylla*, JN934735, JN934777; *Paraboea trisepala*, JN934736, JN934778; *Paraboea umbellata*, JN934737, FJ501317; *Paraboea velutina*, JN934738, JN934780; *Paraboea verticillata*, JN934739, JN934781; *Paraboea vulpina*, JN934740, JN934782; *Paraboea xiangguiensis*, MF358728, MF358711.

**Outgroups**: *Middletonia evrardii*, KU203885, KU203790; *Middletonia monticola*, KU203884, KU203789; *Middletonia multiflora*, MU203886, MU203791; *Isometrum farreri*, JF697585, HQ327464; *Kaisupeea herbacea*, FJ501459, FJ501309; *Ornithoboea arachnoidea*, JN934709, FJ501312; *Ornithoboea wildeana*, JN934710, JN934752.
